# A multisite study of performance drivers among institutional review boards

**DOI:** 10.1017/cts.2017.8

**Published:** 2017-07-24

**Authors:** Michael Caligiuri, Karen Allen, Nate Buscher, Lisa Denney, Cynthia Gates, Kip Kantelo, Anthony Magit, Rachael Sak, Gary S. Firestein, John Fontanesi

**Affiliations:** 1 School of Medicine, University of California San Diego, La Jolla, CA, USA; 2 School of Medicine, University of California Irvine, Irvine, CA, USA; 3 University of California Biomedical Research Acceleration, Integration & Development (UC BRAID), San Francisco, CA, USA; 4 School of Medicine, University of California San Francisco, San Francisco, CA, USA; 5 School of Medicine, University of California Davis, Davis, CA, USA; 6 School of Medicine, University of California Los Angeles, Los Angeles, CA, USA

**Keywords:** Common metrics, Institutional Review Board, clinical trials, best practices

## Abstract

**Introduction:**

The time required to obtain Institutional Review Board (IRB) approval is a frequent subject of efforts to reduce unnecessary delays in initiating clinical trials. This study was conducted by and for IRB directors to better understand factors affecting approval times as a first step in developing a quality improvement framework.

**Methods:**

807 IRB-approved clinical trials from 5 University of California campuses were analyzed to identify operational and clinical trial characteristics influencing IRB approval times.

**Results:**

High workloads, low staff ratios, limited training, and the number and types of ancillary reviews resulted in longer approval times. Biosafety reviews and the need for billing coverage analysis were ancillary reviews that contributed to the longest delays. Federally funded and multisite clinical trials had shorter approval times. Variability in between individual committees at each institution reviewing phase 3 multisite clinical trials also contributed to delays for some protocols. Accreditation was not associated with shorter approval times.

**Conclusions:**

Reducing unnecessary delays in obtaining IRB approval will require a quality improvement framework that considers operational and study characteristics as well as the larger institutional regulatory environment.

## Introduction

Institutional Review Boards (IRBs) are undergoing a major paradigm shift as key stakeholders voice concerns about the economic and human costs of federally mandated regulatory oversight of human subjects research. Since their inception, IRBs were assigned a “gatekeeper” role, ensuring that human research adheres to accepted ethical and regulatory practices. As the workload and the complexity of academic IRBs increases to meet the research and development pressures placed on university faculty, the ability of individual IRBs to function efficiently has been more closely scrutinized [1].

Two recent reports of IRB processes have explored how the variation between institutional workflow and trial characteristics affected the time required to receive approval. Drezner and Cobb [2] evaluated 2 IRB approval time surveys. The first study, involving 33 sites and 425 approved protocols, found that operational characteristics such as the number of IRB committees per site, meetings per month, IRB staffing levels as well as study patient populations (e.g., pediatric, other vulnerable populations, oncology) influenced time to approval. A follow-up survey—expanded to 43 sites, over 1400 protocols—revealed that high volume and experienced staff were associated with faster approval times.

A second group [3] examined administrative processes and timing of approvals for 218 trials from a national cancer center and affiliates. Contrary to expectations, the authors found IRB approval accounted for <25% of the total start-up time. Moreover, their analysis revealed that much of the additional time was related to ancillary approvals from entities such as Radiation Safety Committees and Medicare Coverage Analysis. Given these findings, it is important to acknowledge that focusing solely on IRB performance, in isolation from these other entities, might not reduce the time required to open clinical trials. In addition, despite currently being neglected from methodological discussions, speed alone should not be the sole metric considered when seeking to improve IRB performance; in some cases, protocols require revision and delays could be entirely appropriate.

To obtain a new perspective on IRB processes and efficiency, the IRB directors and the University of California, Biomedical Research, Acceleration, Integration, and Development (UC BRAID) collaborated on a project to define and assess key metrics for IRB efficiency and set the stage for quality improvement efforts within and across 5 California campuses of University of California (UC). The IRB directors identified the main operational and study characteristics to be analyzed based on their collective experience. Each of the variables selected was then operationally defined (including supporting examples) to ensure data harmonization and interinstitutional comparability.

The goals are were to identify (1) operational and study characteristics that affect administrative, committee, and total IRB approval times, (2) compare data between the 5 UC campuses for “lessons learned” that can identify practices associated with shorter approval times, and (3) begin developing a high-level quality improvement assessment framework.

## Materials and Methods

### Study Design

This study examines the relationship between the operational characteristics (the “institutional capacity”) of 5 UC IRB offices, the attributes of clinical trials (“nature of the task”) involving Food and Drug Administration (FDA)-regulated products and the time required to obtain IRB approval (“outcome”). Operational characteristics included frequency of IRB meetings, staffing ratios, volume of approvals, and type of accreditation. To calibrate performance drivers across campuses, analysis for this segment of the study was restricted to phase 3 multisite studies.

Study attributes included phase, funding source, authorship, and types of ancillary reviews, among others. Approval times were operationally defined as:

- The number of days between initial submission of a research approval application to the IRB and the submission being sent to Committee (Administrative Review),
- The number of days between initial IRB review and full Committee approval (Committee Review),
- The number of days between initiation and full IRB approval (total duration).

Eligible trials met all of the following three criteria: (1) they were designed to evaluate the safety, tolerability, or efficacy of an FDA-regulated device or drug; (2) they were received by an IRB on or after January 1, 2014; and (3) they obtained full IRB approval on or before December 31, 2014. Table 1 presents a detailed list of all data elements used in this analysis.

**Table 1:** Data collected for analysis

Outcomes			
Days between first received and first reviewed	Days between first reviewed and approved	Days between first received and approved	
IRB operational characteristics			
Total number of clinical trials reviewed	Number of committees	AAHRPP accreditation	
Total number of FTEs	Frequency of committee meetings	Proportion of FTE holding CIP certification	
FTE to protocol ratio			
Study characteristics			
Study phase	Federally funded	Investigational device exemption	Biosafety review required
Multisite	Private foundation funded	Oncology/hematology	Conflict of Interest Review required
PI-authored	Investigational drug	Number of ancillary reviews required	Scientific Review Committee
Commercial sponsored	Investigational device	Radiation safety review required	Medicare Billing/Coverage Analysis

AAHRP, Association for the Accreditation of Human Research Protection Programs; CIP, certified IRB professional; FTE, full-time equivalent; IRB, Institutional Review Board; PI, principal investigator.

### Quality Control Process

Data management and quality control adhered to ISO 8000-110:2008 standards and followed CRISP-DM guidelines [4, 5]. Each UC IRB Director received monthly quality control reports on the number of cases submitted that month, the number and type of potential data entry errors for review, and corrected or verified for specific cases. As a result, data entry errors were <0.01% exceeding ISO/ANSI 13606-3:2009 [6].

### Statistical Analyses

#### Data Preparation and Analysis

Continuous data, such as approval times, were evaluated to determine their distribution type and variance homogeneity to help in the selection of the statistical measures. This included Tukey and Grubbs tests to identify statistically significant “outliers” and “extreme” cases. Continuous data not found to satisfy assumptions required for parametric analyses underwent either Mann-Whitney *U* test (with continuity correction), when examining interactions with a single dichotomous variable (e.g., commercially sponsored or PI initiated), or Kruskal-Wallis analysis of variance (ANOVA) when exploring multiple categories (e.g., study phase). Wilcoxon-signed rank test was used when the interactions of interest involved 2 continuous variables such as duration and number of ancillary reviews.

Consistent with current recommendations for conducting quality improvement assessment, we report descriptive statistics using median, standard deviation, upper and lower quartiles, and percent outliers (Tables 2 and 3). The combination of upper and lower quartiles is used in quality improvement to identify how “in control” a particular process is as well as whether there might be subprocesses or special causes associated with “outlier” and “extreme” cases identified by the Tukey and Grubbs tests that require special attention and additional analysis [7, 8].

**Table 2:** Descriptive statistics for three approval times (outcome in days) for 807 qualifying clinical trials for each of the 5 University of California medical campus Institutional Review Boards (IRBs)

Study attributes	Review phase	n	Median	SD	Lower quartile (Q1)	Upper quartile (Q3)	Percent outliers
Phase 1	Admin review	172	26.0	42.2	14.0	36.0	14.5
	IRB review		36.0	68.7	21.5	68.0	9.9
	Total time		75.5	78.4	46.5	110.0	8.7
Phase 2	Admin review	299	21.0	27.1	15.0	35.0	13.4
	IRB review		35.0	57.2	19.0	66.0	7.4
	Total time		66.0	61.4	42.0	104.0	6.4
Phase 3	Admin review	262	23.5	27.5	15.0	37.0	14.1
	IRB review		42.0	52.8	18.0	71.0	7.3
	Total time		71.0	61.1	45.0	113.0	6.5
Device	Admin review	74	28.0	38.0	17.0	49.0	20.3
	IRB review		29.0	51.4	9.0	74.0	4.1
	Total time		73.5	63.4	40.0	111.0	6.8
Multisite	Admin review**	559	24.0	30.3	16.0	38.0	16.1
	IRB review**		33.0	53.1	15.0	57.0	5.7
	Total time**		63.0	62.3	40.0	96.0	6.4
Investigator-initiated	Admin review**	196	29.0	36.1	18.0	49.0	5.6
	IRB review		30.5	53.0	15.5	64.0	1.0
	Total time		74.5	67.1	41.5	107.5	1.0
Commercial sponsored	Admin review**	563	22.0	31.4	14.0	34.0	13.0
	IRB review**		42.0	56.7	20.0	71.0	7.8
	Total time		71.0	63.1	45.0	108.0	6.7
Federally funded	Admin review	135	25.0	32.1	15.0	42.0	6.7
	IRB review		23.0	57.5	6.0	48.0	0.0
	Total time**		60.0	68.8	34.0	83.0	0.7
Private foundation	Admin review**	218	15.0	31.9	14.0	27.0	1.4
	IRB review**		60.0	67.7	35.0	100.0	3.7
	Total time**		84.0	73.1	57.0	123.0	2.3
Investigational drug	Admin review**	679	22.0	31.5	15.0	36.0	13.5
	IRB review		36.0	54.8	19.0	68.0	7.2
	Total time		70.0	62.5	43.0	104.0	6.2
Investigational device, yes	Admin review	89	27.0	34.6	16.0	44.0	16.9
	IRB review		35.0	56.8	15.0	76.0	5.6
	Total time		72.0	62.2	44.0	112.0	9.0
Hematology, oncology, yes	Admin review*	336	21.0	38.4	14.0	36.0	16.1
	IRB review		39.0	60.2	19.0	72.0	7.4
	Total time		75.0	66.9	44.0	116.0	6.3

* p≤0.01. ** *p*≤0.001.

**Table 3:** Descriptive statistics related to regulatory aspects for three approval times (outcome in days) for 807 clinical trials for University of California Institutional Review Boards (IRBs)

Study attributes	Review phase	n	Median	SD	Lower quartile (Q1)	Upper quartile (Q3)	Percent outliers
Radiation safety	Admin review	410	22.0	34.5	14.0	37.0	15.4
	IRB review		40.0	64.0	19.0	71.0	8.3
	Total time		75.0	68.2	45.0	116.0	6.3
Biosafety review	Admin review*	118	28.0	34.6	15.0	49.0	22.9
	IRB review		38.0	60.7	21.0	68.0	6.8
	Total time*		81.5	67.0	48.0	119.0	7.6
Conflicts of interest	Admin review	47	22.0	26.2	15.0	30.0	12.8
	IRB review		57.0	54.6	21.0	90.0	8.5
	Total time		85.0	56.2	50.0	118.0	10.6
Scientific review	Admin review**	457	27.0	36.6	15.0	48.0	21.0
	IRB review		36.0	58.1	19.0	68.0	7.7
	Total time**		77.0	65.8	47.0	116.0	7.7
Coverage analysis	Admin review**	691	23.0	32.3	15.0	38.0	15.5
	IRB review		37.0	59.1	19.0	69.0	7.4
	Total time*		73.0	65.5	45.0	110.0	7.2

* p≤0.01. ** *p*≤0.001.

## Results

Eight hundred seven protocols met criteria for inclusion into this study of which 205 were phase 3 multisite studies. Results presented in this section are restricted to variables with statistically significant results ≤0.01.

### Operational Characteristics

#### Operational Characteristics and Administrative Review Times

Staff workload, as measured by the number of protocols per FTE (*p*=0.0047) negatively impacted administrative review time whereas staff training, as measured by percent of certified IRB professional staff (*p*=0.0029) had a positive effect. Interestingly, there was also suggestion of a *queuing effect* where more frequent IRB Committee meetings was associated with shorter administrative review times (*p*=0.0249).

#### Operational Characteristics and Committee Review Times

Dramatic differences in median time to complete reviews between individual committees were observed within each university. Of the 15 individual IRB committees contributing to this study, 3 accounted for 82% of all the committee review outliers though only represented 30% of the studies included in the analysis (*p*=0.019). The median committee review time for these 3 committees was 57 days (interquartile=34–71) compared with 28.5 (interquartile=14–55.5) for the other 12 committees. The only other operational characteristic affecting committee review times was the frequency of meetings (*p*=0.0466).

#### Operational Characteristics and Total Times for Approval

Operational characteristics affecting total approval times reflect those of administrative and committee review times. Specifically, higher staff workload was associated with longer total approval times (*p*=0.0026) while certified IRB professional-certified staff were associated with shorter total approval times (*p*=0.0006).

### Study Characteristics

#### Study Characteristics and Administrative Review Times

The most striking influence on time required to complete administrative reviews was the number and type of ancillary reviews by other regulatory committees (*p*=0.0063). In particular, Biosafety Review (median=28, SD=34.6, interquartile=15–49), Scientific Review (median=27, SD=36.6, interquartile=15–48), and Coverage Analysis (median=23.0, SD=32.3, interquartile=15–38) were most notably associated with longer approval durations. Indeed, all phase 3 multisite administrative review outliers (100%) implicated Coverage Analysis as a driver. In addition, investigator-initiated studies (median=29, SD=36.1, interquartile=18–49) and multisite studies (median=24, SD=53.1, interquartile=16–38) also required significantly longer time for administrative review.

Conversely, private foundation funded (median=15, SD=31.9, interquartile=14–27) and commercially sponsored studies (median=22, SD=31.4, interquartile=14–34) required significantly less administrative review time, as did studies involving investigational drugs (median=22, SD=38.4, interquartile=15–36) or oncology studies (median=21, SD=38.4, interquartile=14–36).

#### Study Characteristics and Committee Review Times

The number, rather than the type of ancillary reviews, associated with a given study proved to be the dominant influence on Committee Review times (p=0.0027). Funding source also affected the time required by an IRB Committee to approve a new proposal, often in the opposite direction from that found with administrative review times. For instance, private foundation funded (median=60, SD=67.7, interquartile=36–100) and commercially funded studies (median=42, SD=56.7, interquartile=20–71) required significantly longer committee time. Yet, federally funded (median=23, SD=57.5, interquartile=6–48), PI initiated (median=30.5, SD=53.0, interquartile=15.5–64), and multisite studies (median=33, SD=53.1, interquartile=15–57) required less committee review time.

#### Study Characteristics and Total Times for Approval

**Fig. 1:**
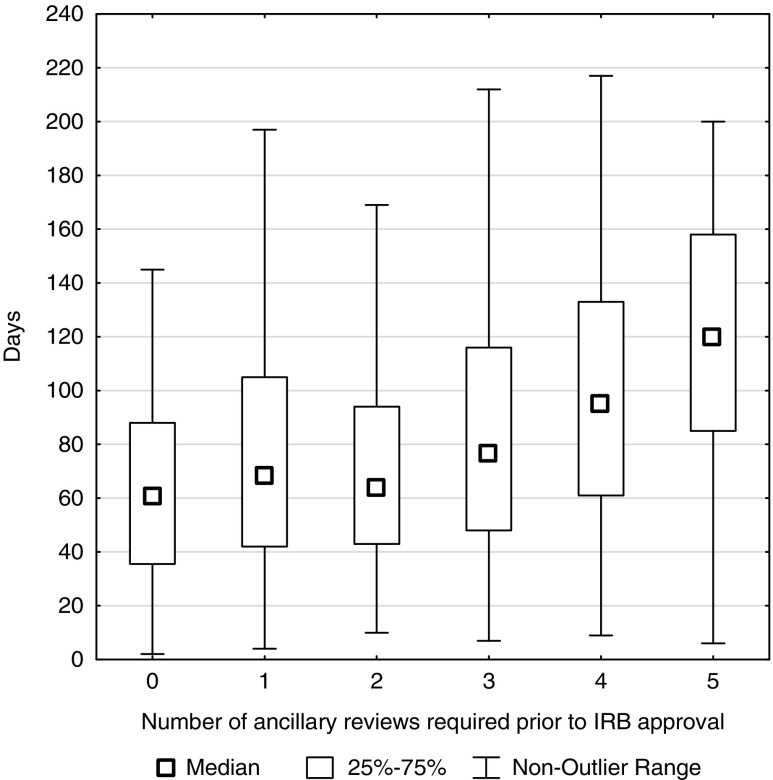
Association between number of ancillary reviews and overall time to Institutional Review Board (IRB) approval. Account name: CITY DRAGON CHINESE INC. Customer account no: 8030771326.

Consistent with both administrative and committee review times, the number and types of ancillary reviews significantly influenced total review time or duration. Biosafety Review (median=81, SD=67, interquartile=48–119), Scientific Review (median=77, SD=65.8, interquartile=47–116), and Coverage Analysis (median=73, SD=65.5, interquartile=45–110) again feature prominently but total number of reviews (*p*≤0.0001) dominated as a mediator of total time required to receive IRB approval (Fig. 1).

Funding source also was related to total approval time, with federally funded studies (median=60, SD=68.8, interquartile=34–83) approved significantly faster than privately funded studies (median=84, SD=73.1, interquartile=57–123). Finally, multisite studies (median=63, SD=62.3, interquartile=40–96) also required significantly less time to receive IRB approval.

## Discussion

The present study of over 800 clinical trials is one of the largest multisite efforts to understand variables that affect IRB performance adding to the emerging body of literature [2, 3, 9–13]. Uniquely, this study was created by and for IRB directors from multiple campuses working under the same institutional policies in meeting those institutional polices as efficiently as possible. This allowed us to not only focus on issues identified as involved with the regulatory process but also to compare procedures and begin formulating best practices. The findings both support previous published results as well as identify new factors affecting IRB approval time.

Consistent with previous findings [2, 3], ancillary reviews increased the time required to receive IRB approval. Three campuses reported only 15% of their clinical trials required 3 or more ancillary reviews whereas 50% of the clinical trials at the other 2 campuses had 3 or more ancillary reviews. The median time to receive IRB approval between the 2 groups was 58 and 85 days, respectively. The difference between campuses was true even for phase 3 multicenter studies, suggesting a difference in policy interpretation at the 2 groups of campuses. This underscores the importance of not simply writing policies but also providing clarifying use cases to help calibrate decisions about when the IRB can review and make recommendations that might otherwise be the responsibility of a separate ancillary committee.

A new factor identified is the impact of workload (number of protocols per FTE) on administrative review time. There was no attempt to identify optimal ratios in the tradeoff between cost of IRB employees and the opportunity costs of delayed IRB approval.

The study highlights the value of frequent feedback in improving performance in 2 different domains: data quality and response time. Using ISO/ANSI 13606-3:2009 standards to provide monthly feedback and data entry errors reduced the rate of errors from 6% during the first month to <0.01% by the ninth month. Equally striking was the reduction in median total duration from 103 days during the first quarter of this study to a median of 62 days by the third quarter of data collection. There is enormous potential for improvement by including quality improvement such as Statistical Process Control and Pareto Charts to monitor both durations and factors associated with “outlier” rates. At this time, and within this consortium, each of the campuses is individually applying “lessons learned” to their internal operations and the across-campus effort continues under the UC BRAID leadership.

Finally, this study suggests that while there are categories of study and operational characteristics that impact approval times, the pattern of critical variables is likely to vary from one IRB to the next. Rather than focus on a single outcome measure such as total duration time, it is more productive to develop a quality improvement framework that individual IRBs can use to identify challenges at their respective campuses. Doing so would permit the development of a *common problems-common solutions* matrix. Such a framework would minimally be composed of 4 dimensions (see Table 4).

**Table 4:** Suggested analytic framework for Institutional Review Boards quality improvement efforts

Dimension	Potential contributors	Potential solutions
Staffing workload	Inadequate staffing Wrong mix Training Inadequate infrastructure	Benchmarking Develop business case Identify key content knowledge and work ethics Explore options for supportive technology
Committee workload	Meeting frequency Volume and types of reviews X meeting X committee member Member training/understanding Member knowledge and expertise Committee dynamics Infrastructure	Benchmarking Feedback/policy/education Explore options for supportive technology
Application quality	Researcher knowledge of current regulatory requirements	Training/concierge service Supportive templates and use cases
Regulatory environment	Number of other regulatory units	Institutional policy on sequential or parallel processing Harmonized requirements and application forms Supportive templates and use cases

The first, most obvious and seldom mentioned in the literature, is IRB staff workload. This domain has 4 major components:

1. Staffing levels for the volume of work,
2. the mix of trained analysts and support staff,
3. the adequacy of infrastructure such as electronic tracking systems, and
4. the skills and training of the staff.

The second potential dimension for assessment is committee-to-committee variation in workflow, expertise and guidance provided by staff and the chairperson. For example, 3 of the 13 Committees included in this study accounted for the majority of “outlier” cases even though they reviewed only 30% of the protocols. Potential causes include:

1. Committee workload (the number of new, renewal, deferred, addendum, violation, adverse event, and safety reports assigned to each committee member per meeting);
2. The knowledge and expertise of committee members relative to the content of the IRB application (e.g., a committee member—unfamiliar with standard of care for a given discipline—may not be comfortable in determining risk levels and will ask for additional information from an applicant); and
3. The internal dynamics of a committee. There may be times in which competition develops between committee members to identify errors in the study design.

The third dimension is the quality of submitted protocols. Regulations affecting IRB determinations are both dynamic and complex. Timing and context matter: what may have been considered a complete and adequate application 5 years ago might no longer meet regulatory review requirements. Conversely, advances in complex adaptive clinical trial designs or in personalized medicine research pose challenges to current guidelines. Drivers of these issues are:

1. Current regulatory knowledge of the investigator submitting a protocol,
2. The degree to which that investigator is able to articulate the risk and benefits of the proposed protocol.

The last dimension is the regulatory environment within which the IRB functions. This analysis corroborates previous studies [3] suggesting that much of the delay in IRB approval processes is driven by ancillary reviews from other regulatory units such as Medicare Coverage Analysis or Radiation Safety. Examples of such issues include:

1. Campus policies determining if ancillary reviews are conducted in parallel or in sequence to IRB review,
2. The degree of communication and harmonization between the different regulatory units,
3. The overall culture of the larger regulatory environment within which the IRB functions.

The present study has a number of potential limitations. First there was no attempt to define what quality of IRB administrative or committee review means. The rapid risk-benefit analysis for a study does not mean it is done well. This is an essential element of analysis that must be considered in any future iteration. Second, it is possible that important trial characteristics such as enrollment of vulnerable populations or research involving Veterans Administration (VA) facilities have been overlooked. Third, we did not attempt to control for the uneven volumes of human subjects research activity across the 5 campuses. These differences may operate to influence approval times in yet unknown ways. The 807 clinical trials that met criteria for inclusion into this study represented only a portion of the human subjects research portfolio at the participating UC campuses. As such, review and approval times may not reflect the levels of efficiencies for a given campus. Finally, the time to approval analyzed in the present study did not include the time from approval to the time of release nor enrollment of the first patients into a trial, an important factor considered by many in industry to be representative of an institution’s regulatory efficiency.
